# Cannabinoid‐Induced Hyperphagia is Mediated by Increased Meal Frequency and the Orexin‐1 Receptor in Male Rats

**DOI:** 10.1002/prp2.70171

**Published:** 2025-09-05

**Authors:** Magen N. Lord, Grace C. Madu, Ana L. Loera‐Lopez, Alexander P. Aaron, Jessica Lin, Emily E. Noble

**Affiliations:** ^1^ Department of Nutritional Sciences University of Georgia Athens Georgia USA; ^2^ Department of Neuroscience Georgia State University Atlanta Georgia USA; ^3^ Department of Psychology University of Georgia Athens Georgia USA

**Keywords:** CB1, c‐Fos, foraging behavior, hypocretin, locomotor activity, meal patterns, microstructure

## Abstract

Exogenous cannabinoids have long been known to promote eating. However, the underlying mechanisms have not been completely elucidated, which is critical to understanding their utility. The orexin/hypocretin (OH) system of the lateral hypothalamus (LHA) has known anatomical, biochemical, and physiological interactions with the endocannabinoid system, and has an established role in promoting appetitive behavior; yet, it is still unknown if the OH system mediates food intake following cannabinoid administration. Herein, we validated an oral method of cannabinoid receptor agonist, CP55940, administration via gelatin‐based edibles, showing that voluntarily consumed cannabinoid‐containing edibles produce acute hyperphagia via an increase in meal number in male rats. Following cannabinoid administration, rats displayed an upregulation in the immediate early gene c‐Fos in OH neurons compared to vehicle‐treated animals. We further employed a within‐subjects design to investigate whether orexin‐1 (OX1) receptor signaling was necessary for cannabinoid‐induced hyperphagia by coadministering a subeffective dose of an OX1 receptor antagonist, SB334867, with the cannabinoid‐containing edible. Data were collected from metabolic monitoring cages, simultaneously capturing chow intake, locomotor activity, and metabolic variables. Results showed that the OX1 receptor antagonist blocked cannabinoid‐induced hyperphagia and the transient increase in locomotor activity following cannabinoid administration. Furthermore, both the edible cannabinoid receptor agonist and the OX1 receptor antagonist individually reduced energy expenditure several hours following administration. Taken together, we conclude that the OX1 receptor is required for the hyperphagic response to exogenous cannabinoid administration.

## 1 Introduction

Cannabinoid‐based therapeutics are an emerging market, but despite the well‐known hyperphagic effects associated with cannabinoid use, we still do not fully understand how exogenous cannabinoids work in the brain to promote hyperphagia. In order to fully realize their medicinal value, a deeper understanding of how isolated cannabinoids have their intended effects and their unintended side effects is warranted. Cannabinoids act via a vast distribution of cannabinoid receptors throughout the body, with the majority of cannabinoid type‐1 (CB1) receptors located in the central nervous system (CNS) [1]. CB1 receptors are the most widely distributed G‐protein–coupled receptor in the CNS, and relevant to this work, are expressed in the reward‐related limbic structures and the hypothalamus [2].

One of the established effects of CB1 receptor agonists is an increase in eating behavior [3, 4, 5, 6, 7], and the hypothalamus is chief among several brain regions that orchestrate eating behavior [8]. CB1 receptors distributed among subpopulations in the hypothalamus are known to contribute to eating behavior regulation [9], but some populations have been better studied than others. In the lateral hypothalamus (LHA), CB1 receptors are expressed on orexin/hypocretin (OH) neurons and on the glutamatergic inputs to OH neurons [10]. The OH system consists of two peptides, orexin‐A and orexin‐B, and two receptors, orexin type‐1 (OX1) receptor and orexin type‐2 receptor [11, 12]. Each receptor has been implicated in distinct behavioral processes, with the effects of orexin‐A on the OX1 receptor largely responsible for the food‐seeking properties of the OH peptides [13]. The OH system promotes reward‐seeking behavior, such as palatable food intake, with projections to reward‐related limbic structures as well as other regions of food intake control [14, 15]. Additionally, these projections terminate where the OX1 receptor and the CB1 receptor are coexpressed [16]. Prior research has shown that both a peripherally administered subeffective dose of rimonabant, a CB1 receptor antagonist that crosses the blood–brain barrier, and the CB1 receptor antagonist AM251 administered to the arcuate hypothalamus block the hyperphagic effect of centrally administered orexin‐A [17]. These findings suggest that CB1 receptor signaling is necessary for orexin‐A–induced feeding. However, whether cannabinoid‐induced feeding requires orexin A signaling has not yet been determined. Therefore, this study investigated the necessity of OX1 receptor signaling in cannabinoid‐induced hyperphagia using the cannabinoid receptor agonist CP55940. The OH system plays a well‐known role in food anticipatory activity, spontaneous physical activity, and increased energy expenditure [15, 18]. Therefore, we further analyzed whether the OH system mediates the effects of cannabinoids on activity and energy metabolism. These data are critical to understanding the involvement of the OH system in upregulating food intake following oral cannabinoid ingestion.

Our preliminary research revealed sex differences in the eating patterns of rats following administration of the edible CP55940, with males increasing the number of meals they consumed and females increasing the size of meals they consumed [19]. We continued our research in both sexes, examining OH neuron activation in the lateral hypothalami of male and female rats after CP55940 and observed differential activation wherein males showed an increase in expression of the c‐Fos protein following CP55940 administration, while females showed no difference in c‐Fos expression in OH neurons (unpublished observation). Thus, in this manuscript, we focused only on the effects of the CP55940 interactions with the orexin system in male rodents, while our research into the mechanisms by which CP55940 impacts eating behaviors in females is reported elsewhere [19].

## 2 Methods

### 2.1 Animals

Twenty‐four male Wistar rats (Envigo, Indianapolis, IN, USA) were singly housed on a 12:12 reverse light/dark cycle in a temperature‐controlled vivarium (22°C) with ad libitum access to standard chow (LabDiet 5053, LabDiet, St. Louis, MO, USA) and water except when noted below. Rats were eight to 10 weeks of age upon arrival as determined by the supplier. Rats were handled and weighed daily. All procedures were approved by the Institute of Animal Care and Use Committee at the University of Georgia (Athens, GA, USA; protocol number A2022 06–035‐A12).

### 2.2 Drugs

The cannabinoid receptor agonist CP55940 (Item No. 13608) and the OX1 receptor antagonist SB334867 (Item No. 19145) were obtained from Cayman Chemical (Cayman Chemical, Ann Arbor, MI, USA). CP55940 was administered as a gelatin‐based edible as previously described [19]. Briefly, edibles are composed of coconut oil, lecithin, Jello, potassium sorbate, gelatin, and distilled water. The cannabinoid was dissolved in coconut oil and lecithin at 0.18 mg/mL and diluted with Jello, potassium sorbate, and gelatin to 0.06 mg/mL. Vehicle edibles are of identical composition without the addition of the cannabinoid drug. Edible molds are 2 mL, and therefore, all edibles were delivered at 2 mL/kg to deliver 0.12 mg/kg according to their weight. This dose was selected based on pilot studies to determine a hyperphagic dose of CP55940.

SB334867 was first dissolved in 100% DMSO, then diluted in cyclodextrin in distilled water that was slightly heated (37°C) to 1 mg/mL. The final concentrations were 10% cyclodextrin and 4% DMSO in distilled water, and this was the composition of the vehicle. 3 mg/kg SB334867 and its vehicle were delivered at 3 mL/kg intraperitoneally (i.p.).

### 2.3 Experiment 1: Standard Chow Intake and Meal Patterns Following Edible CP55940 or Vehicle

Sable Systems Promethion Core metabolic and phenotyping system was used (4826 Rat cage; Sable Systems International, North Las Vegas, NV, USA) to measure male rodents' standard chow intake and meal patterns following edible CP55940 or vehicle, validating this method of cannabinoid administration as a model of cannabinoid‐induced hyperphagia in male rats. Sable Systems automatic recording of behavioral events allows for minimal disturbance to the animal, enabling more accurate data with high temporal resolution. Data were recorded over 24 h, with a measurement being recorded each time the animal displaced food from the food hopper. Total food intake was calculated by the hour, and meals were defined as food removal from the hopper greater than 0.2 g and no more than 15 min apart.

Male rats (*n* = 8) were first habituated to the food intake monitoring cages for 7 days. On three of the 7 days, rats were habituated to the vehicle edible (containing no cannabinoid) to alleviate the influence of food neophobia during testing. On the eighth day, chow was removed 1.5 h before the start of the dark cycle. 30 min prior to the start of the dark cycle, rats received either CP55940‐containing edible at 0.12 mg/kg or vehicle edible. At the immediate beginning of the dark cycle, food access was returned to the rats, and food intake data were collected from the monitoring software (Promethion Live Software Platform; Macro Interpreter). After a 72‐h washout period, treatment groups were switched, and the experiment was repeated for a within‐subjects, counterbalanced design.

Animals with more than 3 g of unconsumed chow remaining in the bedding were excluded from analysis (*n* = 1 male). Data analyzed from this first experiment assessed whether an edible formulation of CP55940 affects food intake and if and how meal patterning changes in response to edible CP55940.

### 2.4 Experiment 2: c‐Fos Activity in Orexin/Hypocretin Neurons of the LHA

We assessed c‐Fos expression in orexin neurons following administration of the edible cannabinoid via transcardial perfusion and immunofluorescence staining. Rats (two subsets from cohorts utilized in prior food intake experiments; *n* = 10) received 0.12 mg/kg of edible CP55940 90 min prior to sacrifice to capture the maximum amount of c‐Fos protein expression [5]. Prior to perfusion for immunofluorescence, food was removed 2 h before the start of the dark cycle. Rats were sacrificed during the first hour of the dark cycle and remained fasted prior to sacrifice. Rats were deeply anesthetized with isoflurane and perfused first with ice‐cold 0.9% saline then 4% paraformaldehyde (PFA). Brains were rapidly extracted and allowed to postfix in 12% sucrose‐PFA for 24 h after which they were frozen with isopentane cooled in dry ice and stored at −80°C until sectioning. Brains were sectioned at 30 μm and stored in cryoprotectant at −20°C until immunofluorescence analyses. LHA sections of the perifornical region were chosen according to the Paxinos and Watson rat brain atlas (levels 56–62). Sections were incubated in a 1:1000 dilution of rabbit anti‐orexin‐A (Cat No: H‐003‐30, Phoenix Pharmaceuticals, Burlingame, CA, USA) and a 1:500 dilution of mouse anti‐c‐Fos (Cat No: AB208942, Abcam, USA) primary antibodies and 1:500 donkey anti‐rabbit 647 (Cat No: 711–605‐152, Jackson ImmunoResearch Laboratories, West Grove, PA, USA) and 1:500 donkey anti‐mouse 488 (Cat No: 705–545‐147, Jackson ImmunoResearch Laboratories, West Grove, PA, USA) secondary antibodies tissues were stained as in [20]. Sections were mounted using Prolong Glass anti‐fade mountant (Cat No: P36984, Invitrogen, Eugene, OR, USA) and stored at 4°C to reduce signal loss prior to imaging. Sections were imaged with the LSM 900 confocal microscope at 10× in Zeiss ZEN software and individual images were stitched to visualize the entire LHA of each section. OH + cells and double labeled c‐Fos + OH + cells were semiautomatically counted using ImageJ by researchers blinded to experimental conditions. Researchers were unaware which images were from animals in the vehicle condition and which were from animals in the cannabinoid condition. Our ImageJ protocol was as follows: For each channel, the brightness and contrast were modified to enhance fluorescence and decrease background using a standardized range (OH: 11915–29 313; c‐Fos: 23232–56 511). Background noise was further reduced using the despeckle function, followed by the removal of outliers using size parameters (OH: radius 10; c‐Fos: radius 5). A threshold was then set for each channel on standardized parameters to separate fluorescence from the background (OH threshold: 20007–65 535; c‐Fos threshold: 27100–65 535), and channels were converted to 8‐bit. When applicable, the watershed function was then applied to split closely packed cells that might otherwise be counted as a single object. Using an image merging operation, a third image was generated to reveal the OH + /c‐Fos + colocalization. The analyze particles function then quantified individual ORX + , c‐Fos + , and doubly labeled ORX + /c‐Fos + cells for each image. A representative matched sample from levels 56–62 of seven total sections from the LHA of each male were counted. We calculated the percentage of c‐Fos + OH + cells by dividing the number of c‐Fos + OH + cells by the individual rodent's total OH + cell count to account for individual differences in total OH + cells counted.

### 2.5 Experiment 3: Standard Chow Intake and Metabolism in Response to Edible CP55940 and IP SB334867 or Vehicles

Sable Systems Promethion Core metabolic and phenotyping system was used to simultaneously collect standard chow intake, activity levels, energy expenditure (EE), and respiratory exchange ratios (RER) following exposure to edible CP55940 and the OX1 receptor antagonist SB334867 or their vehicles (*n* = 16). We selected a subeffective dose of SB334867 that was previously shown to have no effect on food intake alone [21]. We employed a within‐subjects, counterbalanced design. Each of the four treatment days was as follows: food was removed 1 h prior to the start of the dark cycle, and an i.p. injection of SB334867 (3 mg/kg) or vehicle was delivered at 3 mL/kg 15 min after the start of the dark cycle. Following i.p. injection of the OX1 receptor antagonist, animals were given edible CP55940 (0.12 mg/kg) or a vehicle edible. 30 min after edible delivery and 1 h after the start of the dark cycle, food was replaced in each cage and data were collected from the monitoring software (Promethion Live Software Platform; Macro Interpreter) for 23 h. This experiment was designed to test if the OX1 receptor is necessary for cannabinoid‐induced increases in food intake and to understand the impact of these drugs on locomotor activity, EE, and RER. Defined in the Sable Systems Data Analysis Guide, EE is measured in kilocalories per hour using the Weir equation and RER is the ratio of VCO_2_ expelled to VO_2_ consumed.

### 2.6 Statistical Analyses

Data were analyzed with Graphpad Prism (Version 10.3.0). Food intake and meal pattern data from Experiment 1 were analyzed using a paired two‐tailed Student's *t*‐test. Immunohistochemical data were analyzed with an unpaired two‐tailed Student's *t*‐test with Welch's correction applied only to the number of OH + neuron counts due to unequal standard error of the mean. Food intake, activity, EE, and RER from Experiment 3 were analyzed with two‐way ANOVA. Post hoc analyses, where necessary, were conducted with uncorrected Fisher's least significant difference (LSD). Outliers were identified with Grubb's test and excluded. Two rats from experiment three were excluded as outliers.

### 2.7 Nomenclature of Targets and Ligands

Key protein targets and ligands in this article are hyperlinked to corresponding entries in http://www.guidetopharmacology.org, the common portal for data from the IUPHAR/BPS Guide to PHARMACOLOGY [22] and are permanently archived in the Concise Guide to PHARMACOLOGY 2019/20 [23].

## 3 Results

### 3.1 Experiment 1: Standard Chow Intake and Meal Patterns Following Edible CP55940 or Vehicle

**FIGURE 1:**
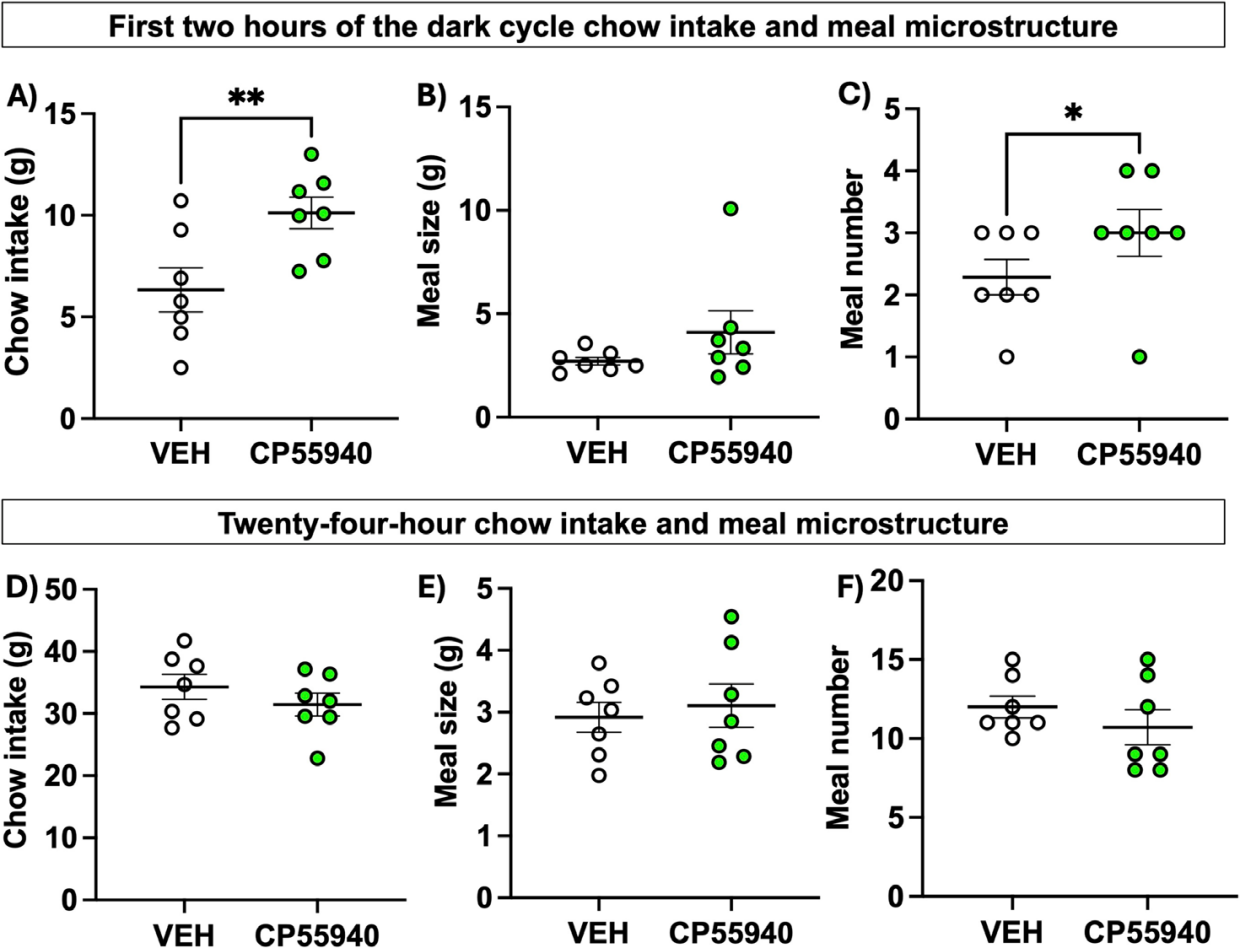
Edible cannabinoid receptor agonist CP55940 acutely promotes eating behavior via an increase in meal number (*n* = 7/treatment). Male rats increased standard chow intake over the first 2 h of the dark cycle (A). Meal pattern analyses revealed hyperphagia was mediated not by an increase in meal size (B) but by an increase in meal number (C). However, over the course of 24 h, there were no differences in overall chow intake (D), meal size (E), or meal number (F). Data were analyzed by paired two‐tailed Student's *t*‐test and are means +/− SEM; *p < 0.05, **p < 0.01.

Over the first 2 h of the dark cycle, male rats given the CP55940‐containing edible increased their intake of chow compared to those given a vehicle edible (Figure 1A; t = 3.765, df = 6, *p* = 0.009). Meal number and meal size were analyzed within this 2‐h window, and we detected that in response to edible CP55940, male rats' meal size remains unaffected (Figure 1B; t = 1.276, df = 6, *p* = 0.25), while meal number increased (Figure 1C; t = 2.5, df = 6, *p* = 0.047) compared to rats given vehicle. However, over the 24‐h period, chow intake (Figure 1D), meal size (Figure 1E), and meal number (Figure 1F) were not significantly different. One rat was excluded due to excess food spillage in the cage bedding, disallowing accurate meal patterning. These data indicate that edible CP55940 acutely increases chow intake via an increase in meal number in the first 2 h after consumption, specifically influencing appetitive behavior in male rats.

### 3.2 Experiment 2: c‐FOS Activity in OH Neurons of the LHA

**FIGURE 2:**
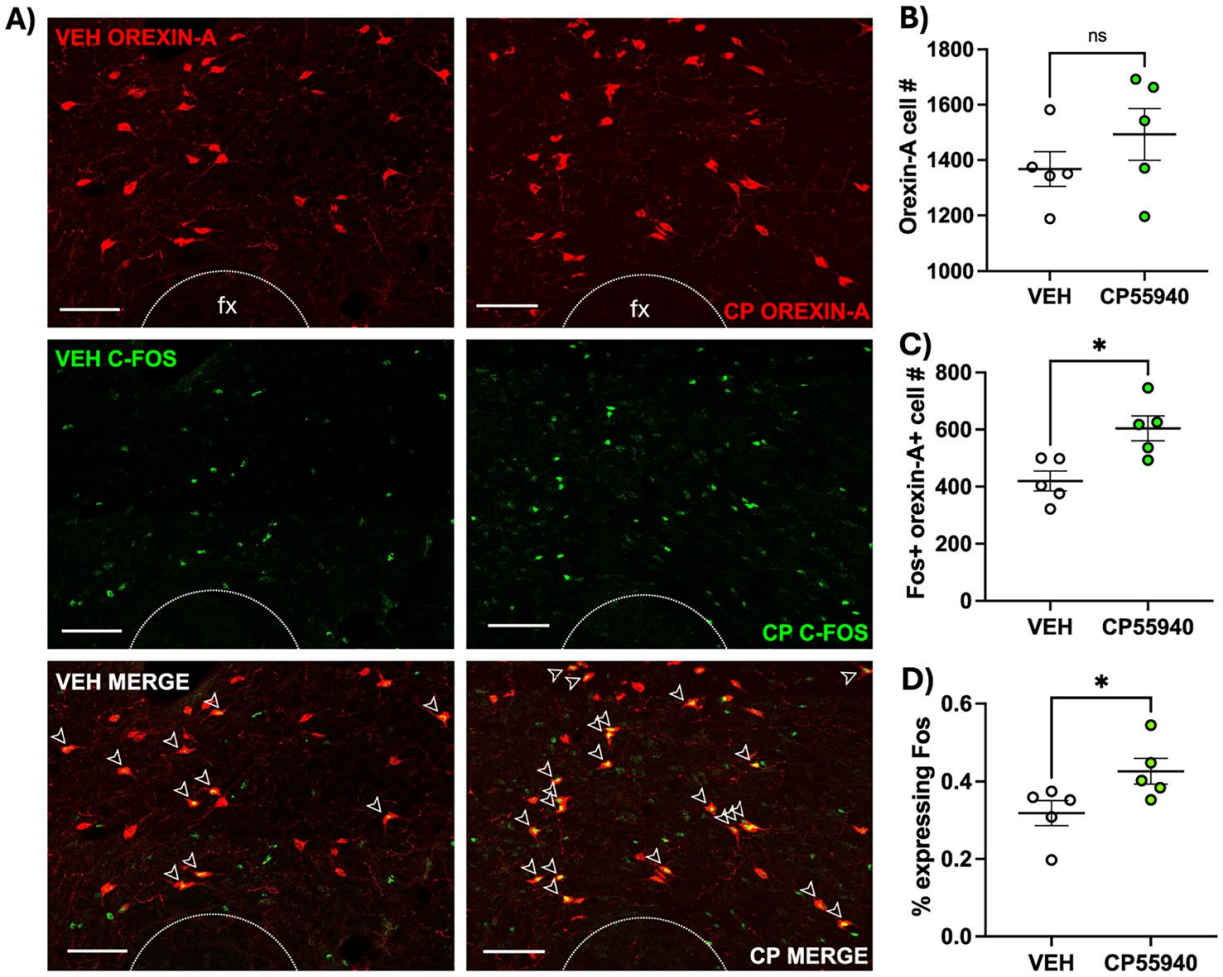
Edible cannabinoid receptor agonist CP55940 increases c‐Fos expression in orexin neurons (*n* = 5/treatment). Representative images from each treatment are shown in (A) with vehicle‐treated in the left column and CP55940‐treated in the right column. There were no differences in total orexin‐A + cell number (B), but there was an increase in doubly labeled c‐Fos + orexin‐A + cells in the cannabinoid‐treated group (C). The percentage of c‐Fos + orexin‐A neurons was calculated for each animal showing that the cannabinoid‐treated group had a higher percentage of orexin‐A neurons expressing c‐Fos compared to vehicle‐treated animals (D). The scale bars are 100 μm. Data were analyzed by unpaired two‐tailed Student's *t*‐test with Welch's correction applied only to the number of OH+ neuron counts due to unequal standard error of the mean and are means +/− SEM; *p < 0.05.

Tissue sections from the perifornical region of the LHA of animals that received edible CP55940 or vehicle 90 min prior to sacrifice were stained for OH and the c‐Fos protein, which is commonly utilized to measure the neuronal response to external stimuli [24]. Subsections of representative images are shown in Figure 2A. Semiautomatic quantification of OH + and c‐FOS + orexin cells via ImageJ reveals no differences in the total number of OH + cells (Figure 2B; t = 1.111, df = 7.015, *p* = 0.3), but a greater number of doubly labeled c‐Fos + OH + cells in the cannabinoid‐treated group, compared to the vehicle‐treated group (Figure 2C; t = 3.306, df = 8, *p* = 0.011). While there were no statistical differences in OH+ cell number, we can account for individual differences in total OH neurons by calculating the percentage of total OH + cells that are doubly labeled. Accounting for individual differences in OH neuron expression, the percentage of orexin cells expressing the c‐Fos protein remained higher in the cannabinoid‐treated group compared to the vehicle group (Figure 2D; t = 2.332, df = 8, *p* = 0.048).

### 3.3 Experiment 3: Standard Chow Intake and Metabolism in Response to Edible CP55940 and IP SB334867 or Vehicles

**FIGURE 3:**
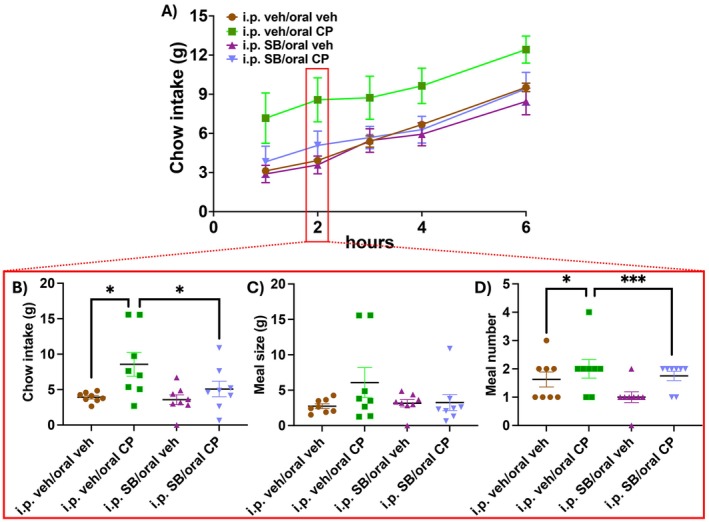
OX1 receptor antagonist SB334867 blocks cannabinoid‐induced hyperphagia (*n* = 8/treatment). Chow intake by the hour is shown in (A) up to 6 h following the return of food access. Two‐way repeated measures ANOVA and post hoc analyses with uncorrected Fisher's LSD of food intake 2 h following food access revealed that CP55940 increased chow intake that was blocked by SB334867 (B). Microstructural meal analysis after 2 h following food access showed no differences in meal size (C), but increased meal number in the cannabinoid‐treated conditions (D). There was a trend toward SB334867 (coadministered with the vehicle edible) decreasing meal number, but this did not reach significance. The red box in A is indicating the time point at which the two‐way cross‐sectional meal analyses were conducted, and the resulting data are displayed in B, C, and D. Data are means +/− SEM; *p < 0.05, **p < 0.01, ***p < 0.001.

Following i.p. injection of the OX1 receptor antagonist SB334867 or i.p. vehicle and administration of edible CP55940 or the edible vehicle, food intake, energy expenditure, RER, and activity levels were simultaneously monitored for 23 h. Six rats were excluded due to nonconsumption of the full dose of the edible, and two rats were excluded as outliers. The first 6 h of chow intake is shown in Figure 3A. 2 h after food replacement (wherein changes in meal patterning were observed in Experiment 1 above), two‐way ANOVA revealed a main effect of CP55940 on food intake (Figure 3B; F (1, 14) = 7.261, *p* = 0.017). Post hoc analyses with uncorrected Fisher's LSD showed that given i.p. vehicle, the cannabinoid‐containing edible increased chow intake compared to vehicle edible‐treated animals (*p* = 0.01). Additionally, SB334867 had no effect on food intake when coadministered with the vehicle edible (*p* = 0.37), while SB334867 coadministered with CP55940 blunted food intake compared to CP55940 coadministration with i.p. vehicle (*p* = 0.029). At the 2‐h mark, meal size remained unaffected by both drug treatments (Figure 3C), but two‐way ANOVA revealed a main effect of CP55940 on meal number (Figure 3D; F (1, 14) = 21; *p* = 0.0004). Post hoc analyses with uncorrected Fisher's LSD showed that edible CP55940 when coadministered with the i.p. vehicle increased meal number (*p* = 0.047), as expected. However, meal number was also increased when CP55940 was coadministered with SB334867 (*p* = 0.001) compared to SB334867 coadministered with the vehicle edible. There was a trend toward SB334867 coadministered with the vehicle edible in decreasing meal number (*p* = 0.08), but this did not reach significance. Food intake following edible CP55940‐i.p. vehicle remained elevated for up to 4 h after administration (Figure S1A; *p* = 0.047), while edible CP55940‐i.p. SB334867 group food intake remained below that of edible CP55940‐i.p. vehicle for up to 6 h (Figure S1B; *p* = 0.038). These differences in food intake and meal microstructure were transient, as over the course of 23 h there were no differences in chow intake between groups (Figure S1C). The acute increase in food intake with no differences in total consumption suggests that there is a compensatory decrease in consumption at some point over the day. In this sample, however, there was no significant decrease in chow consumption at any timeframe analyzed, but one can appreciate from Table S1 that the cannabinoid group did consume nonsignificantly less chow than other groups at almost all other timeframes analyzed beyond 2 h.

**FIGURE 4:**
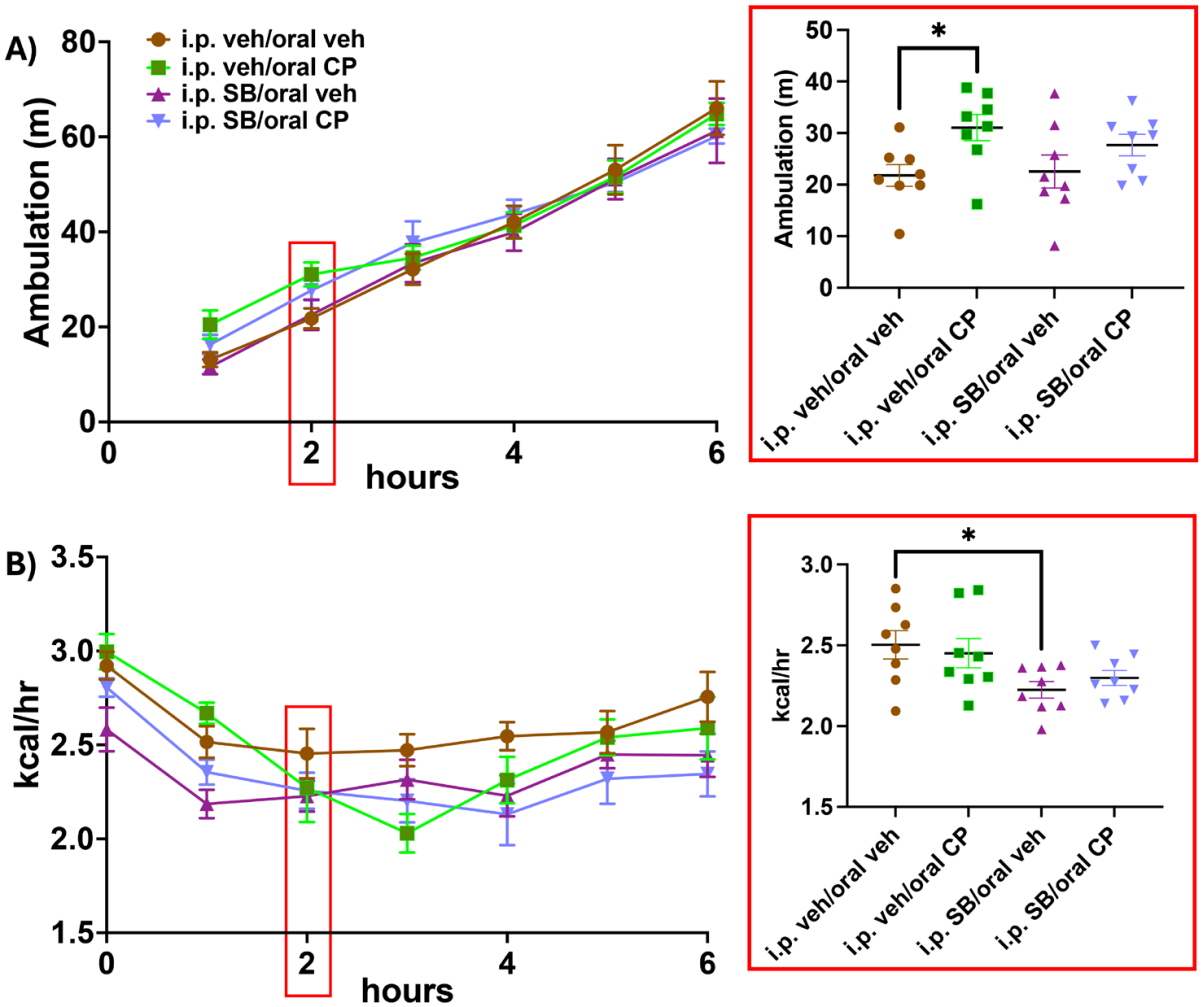
Changes in locomotor activity and energy expenditure following cannabinoid receptor agonist CP55940 and OX1 receptor antagonist SB334867 (*n* = 8/treatment). Locomotor activity in meters is shown over the first 6 h following the return of food access in (A). Two‐way repeated measures ANOVA and post hoc analyses with uncorrected Fisher's LSD of activity 2 h following food access revealed that CP55940 increased ambulation that was attenuated, but not completely blocked, by SB334867 (A, enclosed in red square). Energy expenditure (EE) in kcal/h is shown for up to 6 h over the first 6 h following the return of food access in (B). Two‐way repeated measures ANOVA and post hoc analyses with uncorrected Fisher's LSD of EE 2 h following food access revealed that only SB334867 decreased EE at this time point (B, enclosed in red square). Red boxes on the larger graphs are indicating the time point at which the two‐way cross‐sectional analyses were conducted, and the resulting data are enclosed in the adjacent red square. Data are means +/− SEM; *p < 0.05.

Locomotor activity for the first 6 h following the return of food access is shown in Figure 4A. Coinciding with changes in food intake and meal microstructure, there was a main effect of CP55940 on locomotor activity 2 h after food access was returned (Figure 4A inset; F (1, 14) = 8.157, *p* = 0.013). Post hoc analyses with uncorrected Fisher's LSD revealed that only CP55940 coadministered with i.p. vehicle increased locomotor activity (*p* = 0.021). SB334867 coadministered with CP55940 attenuated the increases in activity at both time points, while having no effect when given with vehicle edible.

EE (kcal/h) for the first 6 h following the return of food access is shown in Figure 4B. 2 h after food replacement at the time that chow intake and locomotor activity were elevated by the edible CP55940, only a main effect of the i.p. injection was detected (Figure 4B inset; F (1, 14) = 5.5, *p* = 0.034), with post hoc analyses with uncorrected Fisher's LSD showing that i.p. SB334867 in combination with the vehicle edible decreased EE (*p* = 0.01). However, further analysis at the 3‐h timepoint following food replacement with two‐way ANOVA showed an interaction between the cannabinoid receptor agonist and the OX1 receptor antagonist on EE during the 3 h following food replacement (Figure S2B; F (1, 14) = 5.72, *p* = 0.032). Post hoc analyses with uncorrected Fisher's LSD revealed that animals given CP55940 coadministered with i.p. vehicle have an overall decrease in EE (*p* = 0.018), and animals given i.p. SB334867 coadministered with the vehicle edible also have decreased EE (*p* = 0.014) compared to the vehicle‐vehicle condition. These data show that each intervention alone, CP55940 and SB334867, decreased EE to a similar degree in the 3 h following food replacement. A main effect of the cannabinoid persisted for 6 h (Figure S3C F (1, 14) = 9.22, *p* = 0.009), with post hoc analyses revealing that EE remained decreased in the edible CP55940‐i.p. vehicle condition (*p* = 0.005) and in the i.p. SB334867‐vehicle edible condition (p = 0.018). The main effect of CP55940 further persisted over the entire dark period (Figure S2A; F (1, 14) = 11.68, *p* = 0.004) with post hoc analyses showing EE was suppressed over the entire dark period in the edible CP55940‐i.p. vehicle condition only (Figure S2D; *p* = 0.005). While the i.p. vehicle‐edible CP55940 group shows a collective decrease in EE over the dark period compared to the vehicle–vehicle condition, there was a nonsignificant elevation in EE in the i.p. vehicle‐edible CP55940 group that only drops below that of the vehicle–vehicle groups after the first 90 min of food access.

Finally, there was a main effect of cannabinoid treatment on RER during the 6 h following food replacement (Figure S3A; F (1, 14) = 15.63; *p* = 0.001). Post hoc analyses with uncorrected Fisher's LSD revealed that CP55940 did not affect RER when coadministered with i.p. vehicle; however, edible CP when coadministered with i.p. SB334867 suppressed RER when compared to the SB334867 antagonist alone (Figure S3B; *p* = 0.001). However, on average, the range of RER between groups was 0.91–0.94, indicating that all groups are oxidizing a mixture of carbohydrates and fat substrates for fuel.

## 4 Discussion

Here, we show that a dual cannabinoid receptor agonist CP55940, orally administered, acutely increases chow intake in male rats via an increase in the number of meals consumed. Elevated cannabinoid‐induced food intake coincides with increased locomotor activity and transiently increased EE, compared to vehicle‐treated animals. EE of cannabinoid‐treated animals is reduced below that of vehicle‐treated animals from the 2 h after food access was returned and up to 6 h. We further show that OH neurons express more c‐Fos following cannabinoid administration, suggesting a higher level of activity in OH neurons with exogenous cannabinoid receptor agonism. Moreover, our data show that OX1 receptor signaling is required for elevated food intake and the increased spontaneous activity observed following edible cannabinoid administration, as the OX1 receptor antagonist SB334867 attenuated these cannabinoid‐induced changes.

The increase we observed in eating behavior following oral CP55940 administration is aligned with numerous reports showing CB1 receptor agonists increase food intake in male rats, including those orally administered [3, 5, 6, 25]. Elevated food intake following a pharmacological intervention is achieved via manipulation of one or more phases of the feeding cycle: Appetition, consumption, and/or satiation [26, 27]. Our microstructural analyses of the eating pattern following edible CP55940 showed that male rats increase their meal frequency rather than meal size, suggesting that the appetitive phase is mainly affected in male rats. Our findings are congruent with data from previous reports showing oral THC administration and whole plant *Cannabis sativa* vapor inhalation increase meal frequency [25, 28]. However, Wheeler and colleagues note a compensatory decrease in meal size following whole plant *Cannabis sativa* vapor inhalation, which we did not observe with the edible CP55940. These differences may be attributed to differences in route of administration, dose of THC, and/or that THC is a partial agonist while CP55940 is a full agonist at the CB1 receptor [28]. The intake behavior observed in our study and by others mimics some of the earliest human data available, showing that after marijuana cigarettes, appetitive “snacking” behavior is elevated in males. Thus, here we show that voluntarily consumed edible CP55940 is a translationally relevant way to investigate the underlying behavioral and neural mechanisms contributing to cannabinoid‐induced hyperphagia. However, after receiving the edible on more than four to five occasions, animals ceased to consume the entire dose and were necessarily excluded from analysis. This is likely due to the association of the effects of CP55940 with the taste of the edible, as others have shown that rodents will consume less of a cannabinoid containing mixture compared to a control formulation [29, 30]. Therefore, future study with voluntary oral cannabinoid administration may seek to build upon this model and look to other successful models of voluntary oral cannabinoid consumption [30, 31].

These food intake data in males further reveal an interesting sex difference in cannabinoid‐induced hyperphagia. Our lab previously demonstrated that, like males, female rats increase their intake of standard chow following edible CP55940 compared to vehicle‐treated females [19]. However, the increase in chow intake in females was due to an increase in meal size rather than meal number, opposite the meal pattern changes in males. As shown above with the increase in meal number in males, we noted that the increase in meal size following cannabinoid administration in females was aligned with the limited literature available in females [32]. Collectively, these data extend the findings of others showing sex differences in the effects of cannabinoids [33, 34] and highlight the importance of sex differences in the design of clinical investigations of how cannabinoids affect eating and other behaviors.

Since its discovery, it has been known that the OH system increases food intake behavior [11], and it was later revealed that these increases were specific to appetitive, food‐seeking behavior [35, 36, 37]. Based on these characteristics, we investigated whether OH neurons were impacted by the hyperphagic dose of CP55940 using the c‐Fos protein as a measure of neuronal activity. Our data reflect that a hyperphagic dose of CP55940 increases c‐Fos expression in the early dark cycle in OH (specifically orexin‐A+) neurons. The literature suggests that differential c‐Fos expression is observed in OH neurons when animals are anticipating a reward, such as a food reward [38, 39, 40]. For example, Cason and colleagues demonstrated increased c‐Fos expression in OH neurons during the extinction phase of a cue‐induced reinstatement protocol [40]. Similarly, Choi and others trained rodents to associate contextual cues with receiving a piece of chocolate, and subsequently sacrificed rats after presentation of the context in the absence of chocolate, showing increased c‐Fos in rodents expecting the chocolate, while differences were absent in rodents not expecting the chocolate reward [39]. In our study, we capitalized on a time period when rodents are already expecting to have their largest meal of the day, the very early dark cycle, when OH neuron activity is elevated based on circadian rhythm [41, 42]. We show that exogenous cannabinoid administration elevates c‐Fos expression in OH neurons above that of an already elevated baseline. These data follow suit with the existing literature showing that c‐Fos expression in OH neurons is selectively elevated with food reward anticipation.

However, c‐Fos data alone is not enough to conclude that the OH system is necessary for cannabinoid‐induced hyperphagia. The food anticipatory properties of OH signaling are largely attributed to orexin‐A ligand binding at the OX1 receptor. The literature on these two closely intertwined systems shows that CB1 receptor antagonists block orexin‐A‐induced eating behavior [17, 43]. Flores and colleagues have investigated the effects of CB1 receptor agonism on orexin type‐2 receptor mediated variables such as hypothermia, antinociception, and anxiety, but not food intake [44]. Furthermore, mice lacking the OX1 receptor have attenuated dopamine release in the nucleus accumbens following THC exposure, and pharmacological blockade of OX1 receptors with SB334867 reduces IV self‐administration of the synthetic cannabinoid WIN55,212–2, suggesting that the physiological reward associated with cannabinoids is partially mediated by the OX1 receptor [45]. We are not currently aware of any studies investigating the involvement of the OX1 receptor in cannabinoid‐induced food intake, preclinical data that may be of interest in the development of cannabinoid‐based therapeutics for appetite regulation. Our data show that a dose of the OX1 receptor antagonist that had no effect on food intake alone blocked the acute CP55940‐induced increases in standard chow intake. However, removal of OX1 receptor signaling does not completely obliterate appetitive behavior produced by cannabinoids, as we found that coadministration of SB334867 with CP55940 did not block increases in meal number. Gonzáles et al. provide convincing evidence that the activity of orexin neurons is immediately suppressed upon contact with food [46], highly consistent with the hypothesis that the neuropeptide specifically promotes appetitive behavior, not within‐meal consumption [35]. However, with the addition of an exogenous cannabinoid that is also promoting appetitive behavior, it stands to reason that blocking the OX1 receptor alone would not be enough to fully extinguish the appetitive drive to initiate another meal in males. Taken together, these data suggest that OX1 receptor signaling is required for the elevated food intake but only partially mediates the appetitive behavior induced by exogenous cannabinoids.

Coinciding with the increases in food intake following cannabinoid consumption, locomotor activity was also increased by CP55940, which is in alignment with an increase in appetitive drive. It is worth noting that at higher doses CB1 receptor agonists have a dampening effect on activity levels [33, 47]. Similar to its impact on food intake, SB334867 also attenuated the cannabinoid‐induced increases in activity, suggesting that these transient increases in activity may be related to the food anticipatory effects mediated by OH. Regarding EE (kcal/h), there was a transient increase in the CP55940 group coinciding with the increase in activity and chow intake. Albeit nonsignificant, the acute increase in activity and EE is in line with a previous report showing these same effects following whole plant cannabis vapor exposure [28]. The transient increase in EE in the CP55940‐treated group was followed by a drop off that lasted up to 12 h. Existing evidence suggests that cannabinoids modulate energy metabolism largely in the periphery of rodents by enhanced lipogenesis [48] and impaired lipolysis [49], and therefore the long‐lasting effect of suppressed EE is consistent with the notion that exogenous cannabinoid administration modulates EE in favor of energy storage [50]. Administration of the OX1 receptor antagonist suppressed average EE to a similar degree as CP55940 and the coadministration of both drugs. Thus, the combination of the two drugs together did not have an additive effect on EE reduction. The reduction in EE following i.p. OX1 receptor antagonist injection is consistent with the well‐established effect of the OH system in stimulating EE through increased arousal [51, 52]. While this dose of SB334867 has been shown to have no effect on locomotor activity [21] as we observed, the dose delivered here effectively reduced EE. Therefore, the suppression of EE observed in cannabinoid‐treated animals is likely not mediated by the central OX1 receptor. This notion is interesting when juxtaposed against our data showing that RER is reduced in animals that received both SB334867 and CP55940, as lower RER indicates increased utilization of fat substrates for energy production. However, the magnitude of this difference was small, with each group on average displaying an RER between 0.91 and 0.94, suggesting that all groups are normally oxidizing a mixture of carbohydrate and fat substrates for fuel.

## 5 Conclusions

Exogenous cannabinoids can enhance the rewarding properties of food, augmenting even bland chow consumption via increased meal number in males, and this behavior is partially mediated by OH neurons and the OX1 receptor. Concomitantly with elevated food intake, locomotor activity was increased by cannabinoid receptor agonism and attenuated by OX1 receptor blockade, which, when taken together with the increases in meal number observed in the cannabinoid‐treated animals, suggests that the cannabinoids act via the OH system to increase meal‐anticipatory locomotor activity. Our data agree with others, suggesting the reduced acceptability of edible cannabinoids in rats with chronic administration. In conclusion, edible cannabinoids act partially via the OX1 receptor to increase appetitive behavior.

## Author Contributions

Magen N. Lord: conceptualization, methodology, formal analysis, investigation, writing, original draft, writing, review and editing, visualization, project administration; Grace C. Madu: investigation; Ana L. Loera‐Lopez: resources; Alexander P. Aaron: investigation; Jessica Lin: investigation; Emily E. Noble: conceptualization, methodology, formal analysis, writing, review and editing, supervision, funding acquisition.

## Ethics Statement

All procedures were approved by the Institute of Animal Care and Use Committee at the University of Georgia (Athens, GA, USA; protocol number A2022 06–035‐A12).

## Conflicts of Interest

The authors declare no conflicts of interest.

## Supporting information


**Figure S1.** Differences in chow intake following cannabinoid receptor agonist CP55940 and OX1 receptor antagonist SB334867. Two‐way ANOVA and post hoc analyses of food intake revealed that chow intake was increased following CP55940 alone and CP55940‐induced hyperphagia was blocked by SB334867 up to 4 h after food access was returned (A). Cannabinoid‐induced hyperphagia was diminished by 6 h after food access was returned, but food intake in the SB334867–CP55940 group was still below that of CP55940 alone (B). These changes were transient, as there were no differences in cumulative food intake over the 23‐h period.
**Table S1.** Noncumulative chow intake following cannabinoid receptor agonist CP55940 and OX1 receptor antagonist SB334867. Each numerical cell represents the average (+/−SEM) grams of standard chow consumed by each condition during the hours indicated (*n* = 8/treatment). Comparing each group within each timeframe with two‐way repeated measures ANOVA did not yield any significant differences. However, one can see that for almost each timeframe beyond 2 h, the cannabinoid group (i.p. veh/oral CP) consumed fewer grams of chow than other groups, indicating a small, nonsignificant suppression of food intake across almost the entire measurement period from 2–23 h after administration.
**Figure S2.** CP55940 suppressed energy expenditure over the dark cycle. Energy expenditure over the dark period is shown in (A). Two‐way repeated measures ANOVA and post hoc analyses of EE 3 h following food access revealed that CP55940 and SB334867 both individually reduced EE, while the combination of the two interventions did not have any additive effect (B), and these differences persisted for up to 6 h (C). Furthermore, cannabinoid treatment individually reduced EE compared to the dual vehicle condition over the entire dark cycle (D). Data are means +/−SEM; *p < 0.05, **p < 0.01.
**Figure S3.** Respiratory exchange ratios following cannabinoid receptor agonist CP55940 and OX1 receptor antagonist SB334867. Average respiratory exchange ratios (RER) are shown in (A) for up to 6 h with arrows indicating the time of i.p. injection, edible administration, and the return of food access. Two‐way repeated measures ANOVA and post hoc analyses of RER revealed that only the dual intervention of edible CP55940 and i.p. SB334867 decreased RER over the 6 ho following the return of food access (B). Data are means +/−SEM; ***p < 0.001.

## Data Availability

The data that support the findings of this study are available from the corresponding author upon reasonable request.
