# Supplementary material for: Cannabinoid‐Induced Hyperphagia is Mediated by Increased Meal Frequency and the Orexin‐1 Receptor in Male Rats
Source: Pharmacol Res Perspect. 2025 Sep 5;13(5):e70171. doi: 10.1002/prp2.70171 (PMC12412231; doi:10.1002/prp2.70171)
Supplement: Supplementary file 1 — Figure S1. Differences in chow intake following cannabinoid receptor agonist CP55940 and OX1 receptor antagonist SB334867. Two‐way ANOVA and post hoc analyses of food intake revealed that chow intake was increased following CP55940 alone and CP55940‐induced hyperphagia was blocked by SB334867 up to 4 h after food access was returned (A). Cannabinoid‐induced hyperphagia was diminished by 6 h after food access was returned, but food intake in the SB334867–CP55940 group was still below that of CP55940 alone (B). These changes were transient, as there were no differences in cumulative food intake over the 23‐h period. Table S1. Noncumulative chow intake following cannabinoid receptor agonist CP55940 and OX1 receptor antagonist SB334867. Each numerical cell represents the average (+/−SEM) grams of standard chow consumed by each condition during the hours indicated (n = 8/treatment). Comparing each group within each timeframe with two‐way repeated measures ANOVA did not yield any significant differences. However, one can see that for almost each timeframe beyond 2 h, the cannabinoid group (i.p. veh/oral CP) consumed fewer grams of chow than other groups, indicating a small, nonsignificant suppression of food intake across almost the entire measurement period from 2–23 h after administration. Figure S2. CP55940 suppressed energy expenditure over the dark cycle. Energy expenditure over the dark period is shown in (A). Two‐way repeated measures ANOVA and post hoc analyses of EE 3 h following food access revealed that CP55940 and SB334867 both individually reduced EE, while the combination of the two interventions did not have any additive effect (B), and these differences persisted for up to 6 h (C). Furthermore, cannabinoid treatment individually reduced EE compared to the dual vehicle condition over the entire dark cycle (D). Data are means +/−SEM; *p < 0.05, **p < 0.01. Figure S3. Respiratory exchange ratios following cannabinoid receptor agonist CP55940 and OX1 receptor [file PRP2-13-e70171-s001.docx]

**Supplementary Figure 1: Differences in chow intake following cannabinoid receptor agonist CP55940 and OX1 receptor antagonist SB334867.** Two-way ANOVA and post-hoc analyses of food intake revealed that chow intake was increased following CP55940 alone and CP55940-induced hyperphagia was blocked by SB334867 up to four hours after food access was returned (A). Cannabinoid-induced hyperphagia was diminished by six hours after food access was returned, but food intake in the SB334867—CP55940 group was still below that of CP55940 alone (B). These changes were transient, as there were no differences in cumulative food intake over the twenty-three-hour period.

| **Treatment** | **0-1 hour** | **1-2 hour** | **2-3 hour** | **3-4 hour** | **4-6 hour** | **6-12 hour** | **12-23 hour (light cycle)** |
| --- | --- | --- | --- | --- | --- | --- | --- |
| **i.p. veh/**  **oral veh** | 3.13 (+/- 0.24) | 0.79 (+/- 0.33) | 1.45 (+/- 0.58) | 1.30 (+/- 0.59) | 2.85 (+/- 0.21) | 9.69 (+/- 0.83) | 7.33 (+/- 0.78) |
| **i.p. SB/**  **oral veh** | 2.89 (+/- 0.67) | 0.69 (+/- 0.45) | 1.87 (+/- 0.59) | 0.48 (+/- 0.35) | 2.52 (+/- 0.70) | 8.99 (+/-1.12) | 6.96 (+/- 0.79) |
| **i.p. veh/**  **oral CP** | 7.17 (+/- 1.93) | 1.40 (+/- 0.90) | 0.16 (+/- 0.16) | 0.91 (+/- 0.60) | 2.78 (+/- 0.58) | 7.28 (+/- 0.80) | 6.78 (+/- 1.20) |
| **i.p. SB/**  **oral CP** | 3.82 (+/- 1.20) | 1.26 (+/- 0.39) | 0.60 (+/- 0.42) | 0.60 (+/- 0.36) | 3.13 (+/- 0.88) | 8.24 (+/- 1.05) | 7.17 (+/- 0.56) |

**Supplementary Table 1: Non-cumulative** **chow intake following cannabinoid receptor agonist CP55940 and OX1 receptor antagonist SB334867.** Each numerical cell represents the average (+/- SEM) grams of standard chow consumed by each condition during the hours indicated (n=8/treatment). Comparing each group within each timeframe with two-way repeated measures ANOVA did not yield any significant differences. However, one can see that for almost each timeframe beyond two hours, the cannabinoid group (i.p. veh/oral CP) consumed fewer grams of chow than other groups, indicating a small, nonsignificant suppression of food intake across almost the entire measurement period from 2-23 hours after administration.

**Supplementary Figure 2: CP55940 suppressed energy expenditure over the dark cycle.** Energy expenditure over the dark period is shown in (A). Two-way repeated measures ANOVA and post-hoc analyses of EE three hours following food access revealed that CP55940 and SB334867 both individually reduced EE, while the combination of the two interventions did not have any additive effect (B), and these differences persisted for up to six hours (C). Furthermore, cannabinoid treatment individually reduced EE compared to the dual vehicle condition over the entire dark cycle (D). Data are means +/- SEM; *p < 0.05, **p < 0.01.

**Supplementary Figure 3: Respiratory exchange ratios following cannabinoid receptor agonist CP55940 and OX1 receptor antagonist SB334867**. Average respiratory exchange ratios (RER) are shown in (A) for up to six hours with arrows indicating the time of i.p. injection, edible administration, and the return of food access. Two-way repeated measures ANOVA and post-hoc analyses of RER revealed that only the dual intervention of edible CP55940 and i.p. SB334867 decreased RER over the six hours following the return of food access (B). Data are means +/- SEM; ***p < 0.001.
